# EfrEF and the Transcription Regulator ChlR Are Required for Chlorhexidine Stress Response in Enterococcus faecalis V583

**DOI:** 10.1128/AAC.00267-18

**Published:** 2018-05-25

**Authors:** Farry J. Li, Kelli L. Palmer

**Affiliations:** a Department of Biological Sciences, The University of Texas at Dallas, Richardson, Texas, USA

**Keywords:** Enterococcus, chlorhexidine

## Abstract

Enterococcus faecalis is an opportunistic pathogen and leading cause of health care-associated infections. Daily chlorhexidine gluconate (CHG) bathing of patients is generally regarded as an effective strategy to reduce the occurrence of health care-associated infections. It is likely that E. faecalis is frequently exposed to inhibitory and subinhibitory concentrations of CHG in clinical settings. The goal of this study was to investigate how the vancomycin-resistant strain E. faecalis V583 transcriptionally responds to and tolerates stress from CHG. We used transcriptome (microarray) analysis to identify genes upregulated by E. faecalis V583 in response to CHG. The genes *efrE* (EF2226) and *efrF* (EF2227), encoding a heterodimeric ABC transport system, were the most highly upregulated genes. *efrEF* expression was induced by CHG at concentrations several 2-fold dilutions below the MIC. Deletion of *efrEF* increased E. faecalis V583 susceptibility to CHG. We found that ChlR, a MerR-like regulator encoded by a sequence upstream of *efrEF*, mediated the CHG-dependent upregulation of *efrEF*, and deletion of *chlR* also increased chlorhexidine susceptibility. Overall, our study gives insight into E. faecalis stress responses to a commonly used antiseptic.

## INTRODUCTION

Enterococci are Gram-positive bacteria and colonizers of the gastrointestinal tracts of humans and animals. Enterococcus faecalis, an opportunistic pathogen, is one of the leading causes of health care-associated infections, including bloodstream infections, surgical wound infections, and urinary tract infections (1). The intrinsic antibiotic resistance of E. faecalis combined with the horizontal acquisition of antibiotic resistance genes often complicates treatment of these infections (2). Vancomycin-resistant enterococci (VRE) are particularly of concern.

Chlorhexidine is a bisbiguanide disinfectant and antiseptic with broad-spectrum antimicrobial efficiency against bacteria. Chlorhexidine gluconate (CHG), a chlorhexidine salt solution, is used for infection control, including whole-body rinsing of patients in intensive care units (ICUs), oral cleansing, and surgical hand washes. The mechanism of action and efficacy of chlorhexidine against bacteria have been studied for decades (3). It is generally postulated that the antimicrobial activity of chlorhexidine stems from its cationic nature. Chlorhexidine attaches to the negatively charged cell envelope, resulting in breakage of the outer leaflet. High concentrations of chlorhexidine severely compromise the cytoplasmic membrane, leading to cell lysis. At lower concentrations, near the MIC, chlorhexidine distorts the cell walls of Gram-positive and Gram-negative bacteria, leading to morphological changes in the cell surface (4). Uptake of chlorhexidine into the cytoplasm causes precipitation of the cytoplasmic components and inhibits ATPase activity (5, 6).

Daily CHG bathing of ICU patients is used to control VRE and other nosocomial infections (7–9). CHG is typically detectable on patients' skin for 24 h postbath (10). However, VRE recover to the prebath density on patients' bodies in less than 24 h (10). This finding indicates that VRE are frequently exposed to inhibitory and subinhibitory CHG concentrations as a result of CHG bathing. Recent E. faecalis isolates from an ICU demonstrated a high prevalence of reduced chlorhexidine susceptibility (11). It is conceivable that extensive use of CHG bathing could select for strains with reduced chlorhexidine susceptibilities.

It is currently not well understood how VRE respond to and tolerate stress from subinhibitory concentrations of CHG. By using microarray analysis, we found that gene expression in E. faecalis V583 is altered after CHG exposure. Of particular interest is that EF2226 and EF2227, which encode the heterodimeric ATP-binding cassette (ABC) transporter EfrEF (12), are the most upregulated genes in E. faecalis V583 upon exposure to CHG. By deletion analysis, we show that *efrEF* expression confers protection from CHG. Further, EF2225 (referred to as *chlR* here), a putative MerR family transcription regulator encoded by a sequence upstream of *efrEF*, mediates the upregulation of *efrEF* in response to CHG exposure.

## RESULTS

### E. faecalis V583 growth kinetics after H-CHG exposure.

E. faecalis V583 is a VanB-type vancomycin-resistant bloodstream infection isolate and model strain for E. faecalis studies (13, 14). The broth microdilution MIC of the commercially available Hibiclens CHG product (H-CHG) for E. faecalis V583 is 9.8 μg/ml, which is within the lowest range of CHG residual concentrations detected on patients' bodies (0 to 18.75 μg/ml) (10).

**FIG 1:**
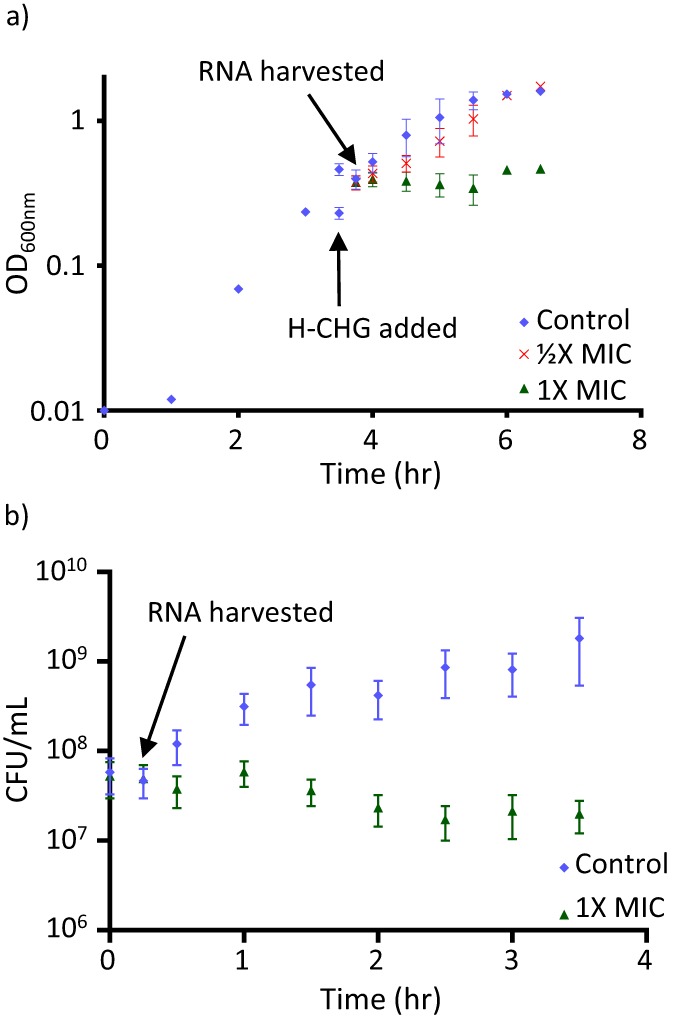
Growth curves. (a) The optical density at 600 nm (OD_600_) is shown on the *y* axis. Mid-exponential-phase E. faecalis V583 cultures (OD_600_, 0.4 to 0.5) were split into fresh, prewarmed medium to achieve different H-CHG concentrations (indicated with the arrow). For all experiments, 1× MIC is the broth microdilution MIC of E. faecalis V583. The time point at which RNA was harvested is also indicated. (b) Viable cell counts (number of CFU per milliliter) for 1× MIC-treated cultures and control cultures are shown. For this curve, the time at which cultures were split is set to 0 h. For both panels, error bars indicate standard deviations from 3 independent experiments.

We assessed the growth of E. faecalis V583 in response to different concentrations of H-CHG by spiking H-CHG into cultures in exponential phase (Fig. 1). We used the H-CHG MIC obtained by broth microdilution as a reference for the amount of H-CHG spiked into the cultures. V583 stops growing after exposure to 1× MIC H-CHG, but cells remain viable (Fig. 1a and b). V583 is initially growth inhibited but recovers to normal growth after exposure to 1/2× MIC H-CHG (Fig. 1a). After 20 h of incubation, the optical density at 600 nm (OD_600_) of cultures exposed to 1× MIC H-CHG was identical (OD_600_, ∼1.8) to that of untreated control cultures.

### *efrE* and *efrF* are highly upregulated in response to H-CHG.

We used custom Affymetrix GeneChips with probes targeting E. faecalis V583 open reading frames (ORFs) (15) to assess the transcriptional response of V583 to H-CHG exposure. Across two independent experimental trials, exposure to 1× MIC H-CHG for 15 min resulted in the ≥4-fold upregulation of 75 genes compared to their expression levels in untreated cells (see Data Set S1 in the supplemental material). Of the 75 genes, 39 (52%) were predicted by the PSORTb (version 3.0) program (16) to encode membrane proteins (Data Set S1). In comparison, only 28.4% of the V583 proteome (884 of 3,112 proteins) is predicted to be membrane proteins (17). This is a significant enrichment for membrane proteins in the H-CHG stress response (χ^2^ test [degrees of freedom = 1, *n* = 3,187] = 18.685, *P* < 0.0001).

**FIG 2:**
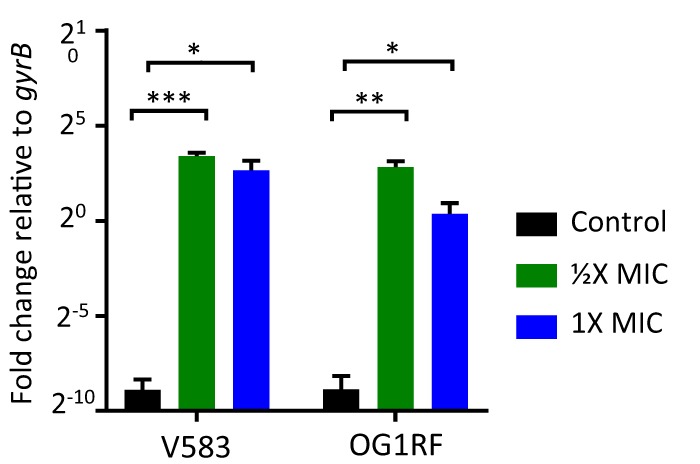
Quantitative RT-PCR confirms H-CHG-dependent upregulation of *efrF*. Primers were designed to amplify ∼600-bp internal regions of *efrF*. RNA was harvested from E. faecalis V583 after 15 min of exposure to no CHG, 1/2× MIC H-CHG, or 1× MIC H-CHG. The gyrase B (*gyrB*) gene was amplified as a control. *, *P* < 0.05; **, *P* < 0.005; ***, *P* < 0.0005.

Of the genes upregulated in response to H-CHG, *efrE* and *efrF* stood out with 286- and 326-fold upregulation, respectively. Semiquantitative reverse transcriptase PCR (RT-PCR) (Fig. S1) analyses confirmed the microarray results for these two genes, and quantitative RT-PCR (Fig. 2) confirmed the upregulation of *efrF* in the presence of H-CHG. We also assessed *efrEF* expression in the vancomycin-susceptible E. faecalis strain OG1RF, which we determined has the same broth microdilution H-CHG MIC (9.8 μg/ml) as E. faecalis V583. In the presence of H-CHG, the *efrEF* orthologs OG1RF_11766 and OG1RF_11767 are upregulated (Fig. 2 and S2).

ABC transporters typically consist of two subunits which function as homo- or heterodimers. A previous study purified EfrE and EfrF and determined that the proteins formed a heterodimer (12). It is therefore likely that *efrE* and *efrF* are cotranscribed and coregulated. To test this, RT-PCR was performed using RNA isolated from E. faecalis V583 grown in the presence of H-CHG. Primers were designed to amplify the 52-bp intergenic region between *efrE* and *efrF*, along with parts of the *efrE* and *efrF* coding regions. The results demonstrate that a transcript containing *efrE* and *efrF* is present in E. faecalis V583 (Fig. S2).

### Deletion of *efrEF* increases H-CHG susceptibility.

**FIG 3:**
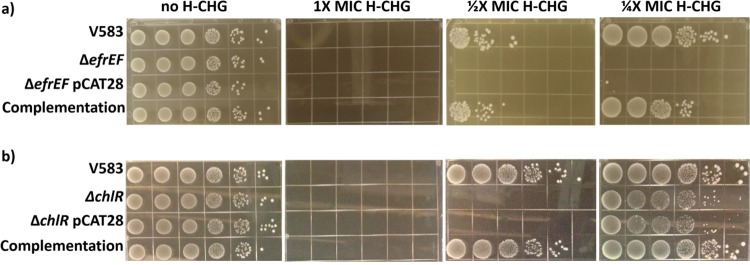
*efrEF* and *chlR* deletion mutants are more susceptible to H-CHG. Overnight cultures were adjusted to an OD_600_ of ∼0.3 and serially diluted in 10-fold dilutions. Ten microliters of each dilution (from 10^−1^ to 10^−6^, from left to right in each image) was spotted on BHI agar supplemented with different concentrations of H-CHG. The images are representative of those from three independent trials. The results of experiments assessing the contributions of *efrEF* (a) and *chlR* (b) to chlorhexidine susceptibility are shown.

To investigate how *efrE* and *efrF* impact H-CHG susceptibility in E. faecalis, we constructed an *efrEF* deletion mutant. Growth on brain heart infusion (BHI) medium was not affected when *efrE* and *efrF* were deleted (Fig. 3a). However, the deletion mutant was more susceptible to H-CHG than the wild-type strain in broth microdilution assays (MIC, 2.4 μg/ml H-CHG) and in agar plate assays (Fig. 3a). We complemented the Δ*efrEF* mutant by expressing *efrEF* from its native promoter on a multicopy plasmid. Complementation restored H-CHG susceptibility to wild-type levels (Fig. 3a).

### ChlR, a MerR family regulator, mediates *efrEF* upregulation in response to H-CHG.

We were interested in how *efrE* and *efrF* are regulated. NCBI Conserved Domains analysis assigns EF2225 to the MerR-like family of transcription regulators. EF2225 is encoded by a sequence upstream of *efrEF* and is divergently transcribed. The layout of EF2225 and *efrEF* is representative of that of a MerR regulon (18, 19). We refer to EF2225 as *chlR* here.

To investigate if ChlR regulates *efrEF* expression, a Δ*chlR* strain was constructed. The Δ*chlR* strain was complemented in *trans* by cloning the complete *chlR* gene and putative promoter into a multicopy vector. The Δ*chlR* mutant strain showed increased susceptibility to H-CHG (Fig. 3b), and its broth microdilution H-CHG MIC (4.9 μg/ml) was half that of the wild-type strain. H-CHG susceptibility was restored to the wild-type level in the complemented strain (Fig. 3b).

**FIG 4:**
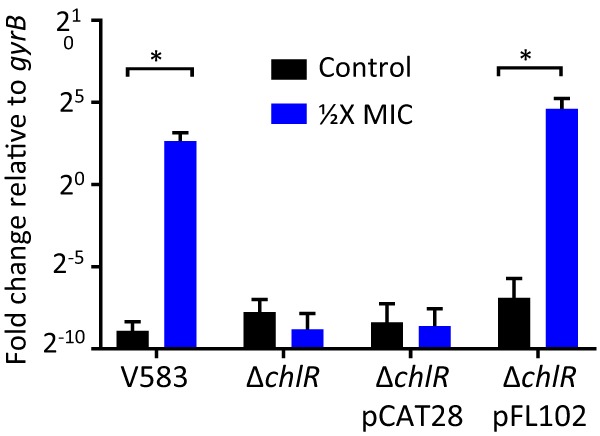
*chlR* is required for H-CHG-dependent upregulation of *efrF*. Quantitative RT-PCR was used to investigate *efrF* expression in cultures of wild-type strain V583, the Δ*chlR* mutant, the Δ*chlR* mutant with the empty complementation vector pCAT28, and the complemented Δ*chlR* strain (Δ*chlR*/pFL102) after 15 min of exposure to 1/2× MIC H-CHG or no H-CHG. The gyrase B (*gyrB*) gene was amplified as a control. *, *P* < 0.05.

To further substantiate the relationship between ChlR and *efrEF*, *efrEF* expression was assessed in the Δ*chlR* and complemented strains by quantitative reverse transcriptase PCR (qRT-PCR). Upon 15 min of exposure to 1/2× MIC H-CHG, the transcription levels of *efrE* and *efrF* in the Δ*chlR* mutant remained the same regardless of the presence of H-CHG, whereas H-CHG induced *efrE* and *efrF* expression in the *chlR* complemented strain (Fig. 4). This result demonstrates that ChlR is required for the upregulation of *efrEF* in response to H-CHG.

**FIG 5:**
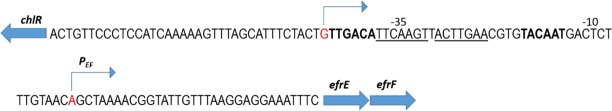
Organization of *chlR-efrE* intergenic region. The organization of the *chlR* and *efrEF* genes is shown (the arrows are not drawn to scale). Transcription start sites (TSS) were detected 33 and 78 bp upstream of the *efrE* ORF. Consensus −35 and near-consensus −10 housekeeping sigma factor promoter sequences upstream of the H-CHG-responsive *efrE* promoter (*P_EF_*) are shown in bold. Putative ChlR binding motifs are underlined. The predicted promoter for the −78-bp TSS is not shown for clarity.

To investigate the *efrEF* promoter, primer extension was used to find the transcription start site (TSS) of *efrE*. Primer extension identified two transcription start sites. Under standard culture conditions in BHI broth, we identified a TSS at −78 bp upstream of the *efrE* ORF. When cells were exposed to H-CHG, an additional TSS was detected at −33 bp upstream of the *efrE* ORF; its corresponding promoter is designated *P_EF_* here. On the basis of this evidence, we propose that the *efrEF* operon has two promoters. One promoter, corresponding to the TSS at −78 bp upstream of the *efrE* ORF, is constitutive and is responsible for basal transcription of *efrEF. P_EF_* is a ChlR-dependent promoter (Fig. 5).

On the basis of existing knowledge of MerR family regulators (18), the *chlR* promoter is predicted to be oriented opposite to *P_EF_*. We obtained inconsistent results across multiple primer extension trials for the *chlR* TSS. The presumptive *chlR* promoter is designated *P_R_* here.

### H-CHG treatment induces ChlR to activate the *efrEF* promoter.

In our microarray trials, the expression of *chlR* was not affected by H-CHG (fold change = 0.3; *P* = 0.57). This evidence indicates that H-CHG may directly or indirectly trigger the activation of ChlR.

**FIG 6:**
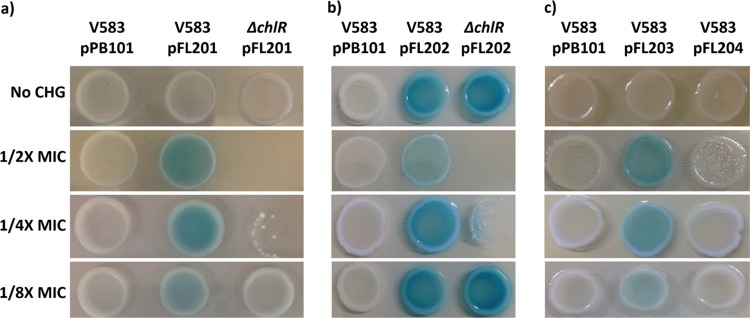
β-Galactosidase assays. Cultures were spotted on BHI agar plates supplemented with X-Gal and different concentrations of H-CHG. Wild-type V583 with pPB101 (promoterless *lacZ*) was used as the negative control for all panels. (a) *efrE* promoter activity (pFL201) in wild-type V583 and the Δ*chlR* strain. (b) *chlR* promoter activity (pFL202) in wild-type V583 and the Δ*chlR* strain. (c) *efrE* promoter activity in wild-type V583 with (pFL204) or without (pFL203) mutation of the 3′ region of the putative ChlR binding motif. The images shown are representative of those from three independent trials.

We performed β-galactosidase assays to assess the responses of the *efrEF* promoter *P_EF_* to H-CHG. Promoter reporter strains were spotted on agar plates supplemented with 5-bromo-4-chloro-3-indolyl-β-d-galactopyranoside (X-Gal) and different concentrations of H-CHG (Fig. 6). As expected, the control strain lacking a promoter for *lacZ*, E. faecalis V583/pPB101 (strain FL101), displayed no detectable β-galactosidase activity in the presence or absence of H-CHG (Fig. 6a). For cultures without H-CHG, *P_EF_* promoter activity was not detected. Subinhibitory concentrations of H-CHG elicited increases in *P_EF_* promoter activity (Fig. 6a). These results demonstrate that H-CHG is required to stimulate *efrEF* promoter activity and that H-CHG concentrations several 2-fold dilutions below the MIC still elicit this response. Conversely, *P_EF_* induction by H-CHG was absent in V583 Δ*chlR* (Fig. 6a). We conclude that ChlR is required for activation of the *efrEF* promoter upon H-CHG exposure.

We constructed a *chlR* promoter reporter, pFL202 (Fig. 4b), to assess the responses of the *P_R_* promoter to H-CHG. Unlike *P_EF_*, *P_R_* was active regardless of the presence or absence of H-CHG. Moreover, *P_R_* was active in V583 Δ*chlR* irrespective of the presence or absence of H-CHG (Fig. 6b).

The *P_EF_* promoter includes a consensus −35 sequence (TTGACA) and a near-consensus −10 region (TACAAT) for binding by a housekeeping sigma factor. The −10 and −35 sequences are separated by 19 bp. This 19-bp spacing is typical for MerR family promoters; MerR regulators recruit RNA polymerase holoenzyme to nonoptimal promoters (18, 19). Unusually, however, the TSS of *P_EF_* occurs 13 bp downstream from the 3′ end of the −10 region. Nonstandard TSS spacing was also observed for the MerR regulation of *merA* in the archaeon Sulfolobus solfataricus (20) but to our knowledge has not been observed for other MerR regulators in bacteria.

Typical MerR-regulated promoters harbor a palindromic MerR binding motif between the −35 and −10 promoter regions (18). Within the *P_EF_* promoter region, we identified a palindromic motif (underlined), TTCAAGTTACTTGAA (Fig. 5), which does not occur elsewhere on the V583 chromosome. Because the 5′ half of the motif lies directly adjacent to the predicted −35 region (Fig. 5), alteration of that sequence may prevent RNA polymerase binding. We modified the 3′ motif from ACTTGAA to CAGCTAC to determine if this motif affects *efrEF* promoter inducibility. H-CHG induction was abolished for the construct with the mutant *P_EF_* promoter (Fig. 6c).

## DISCUSSION

In this study, we performed transcriptomic analysis to identify genes that are differentially regulated when E. faecalis V583 is exposed to H-CHG. The genes *efrE* and *efrF* were the most highly upregulated. We found that *efrEF* and the transcription regulator ChlR are required for the H-CHG stress response in E. faecalis V583. ChlR activates *efrEF* expression in response to H-CHG. These results are consistent with and identify new features of the chlorhexidine stress response in enterococci. The *efrEF* orthologs in a VanA-type VRE strain, E. faecium 1,231,410, were also upregulated in the presence of H-CHG (21). Moreover, sequential subinhibitory H-CHG exposure selected for E. faecium 1,231,410 *efrE* mutations that conferred reduced H-CHG susceptibility (22). Finally, deletion of *efrE* in E. faecalis OG1RF conferred decreased susceptibility to chlorhexidine and pentamidine (23, 24). Our results deepen our understanding of *efrEF* by identifying a transcriptional regulator that is required for the induction of *efrEF* expression in response to H-CHG stress.

Gaps in knowledge about the enterococcal response to chlorhexidine stress remain. Specifically, what does EfrEF transport, and what ligand activates ChlR? These processes are significant because they reduce enterococcal susceptibility to chlorhexidine. Hassan et al. discovered chlorhexidine efflux proteins in Gram-negative bacteria (25, 26), but EfrEF does not belong to this protein family. Overexpression of *efrEF* in Lactococcus lactis conferred enhanced efflux of fluorescent dyes and decreased susceptibility to multiple antibiotics (12). Monitoring of [^14^C]chlorhexidine transport in E. faecalis wild-type and Δ*efrEF* strains would be required to determine whether chlorhexidine is a substrate for EfrEF. Alternatively, EfrEF may transport a metabolite that is required for the cell to survive the stress imposed by H-CHG and other antimicrobials.

ChlR belongs to the MerR regulator family. MerR was first identified as a transcriptional activator of the mercury resistance (*mer*) operon in Gram-negative bacteria. An activated MerR dimer bound at a dyad symmetrical motif sequence in the *mer* promoter region drives a conformational change in DNA that results in induction of *mer* operon expression (18, 19). Generally, MerR family proteins possess two domains: a highly conserved N-terminal DNA binding region and a poorly conserved C-terminal ligand binding region (18, 19). The functionality of the N-terminal region depends on ligand binding by the C terminus. The variable C-terminal sequences of the MerR protein family recognize different ligands, including metals and dyes, and therefore lack amino acid sequence conservation (18, 19). The C-terminal region of ChlR possesses no predicted conserved domains. As shown by our microarray and β-galactosidase reporter analyses, *chlR* expression is not induced by H-CHG. It appears that ChlR requires H-CHG or metabolites associated with H-CHG stress to induce *efrEF* expression. Our study did not determine whether chlorhexidine directly interacts with ChlR. The observation that *efrEF* is upregulated in response to the plasmid postsegregational killing toxin Fst (27) confirms that *efrEF* upregulation is not specific to H-CHG stress. It is unknown whether ChlR mediates the Fst-dependent upregulation of *efrEF*. Identifying the specific ligand of ChlR will be a topic of future studies.

In conclusion, our study provides novel insights into the transcriptomic response of vancomycin-resistant E. faecalis to chlorhexidine. The *chlR* and *efrEF* genes play key roles in E. faecalis survival of H-CHG exposure at concentrations near the MIC. Notably, 1/8× MIC H-CHG activated ChlR-dependent *efrEF* expression; induction at lower concentrations may occur, but we did not test this. In a hospital environment, E. faecalis is likely exposed to subinhibitory concentrations of H-CHG which are not lethal but are sufficient to elicit a transcriptional response. It remains to be determined whether this transcriptional response impacts susceptibility to other clinically relevant antimicrobials.

## MATERIALS AND METHODS

### Bacterial strains and routine molecular biology procedures.

The bacterial strains and plasmids used in this study are listed in Table 1. E. faecalis was routinely cultured in brain heart infusion (BHI) medium with or without agar at 37°C unless otherwise noted. Escherichia coli was routinely cultured in lysogeny broth or agar at 37°C unless otherwise noted. Chloramphenicol was used at 15 μg/ml. PCR was performed with *Taq* polymerase (New England BioLabs) or Phusion (Fisher Scientific). Plasmids were purified using a QIAprep Spin Miniprep kit (Qiagen). Inserts in engineered plasmids were sequenced (Massachusetts General Hospital DNA Core) to ensure that no mutations occurred during cloning. The sequences of the primers used in this study are provided in Table S1 in the supplemental material.

**TABLE 1:** Bacterial strains and plasmids used in this study

Strain or plasmid	Description	Reference or source
Bacterial strains		
E. faecalis strains		
V583	Bloodstream isolate; VanB-type VRE	13
OG1RF	Human oral isolate	33
Δ*chlR*	E. faecalis V583 Δ*chlR*	This study
Δ*efrEF*	E. faecalis V583 Δ*efrEF*	This study
Δ*efrEF*/pFL103	E. faecalis V583 Δ*efrEF* transformed with pFL103	This study
FL101	E. faecalis V583 transformed with pPB101	This study
FL201	E. faecalis V583 transformed with pFL201	This study
FL202	E. faecalis V583 transformed with pFL202	This study
FL203	E. faecalis V583 transformed with pFL203	This study
FL204	E. faecalis V583 transformed with pFL204	This study
Δ*chlR*/pFL102	Δ*chlR* mutant transformed with pFL102	This study
Δ*chlR*/pFL201	Δ*chlR* mutant transformed with pFL201	This study
Δ*chlR*/pFL202	Δ*chlR* mutant transformed with pFL202	This study
E. coli strains		
EC1000	Cloning host; provides *repA* in *trans*; F^−^ *araD139* (*ara ABC-leu*)*7679 galU galK lacX74 rspL thi repA* of pWV01 in *glgB* Km	34
DH5α	Cloning host; F^−^ *endA1 glnV44 thi-1 recA1 relA1 gyrA96 deoR nupG* ϕ80d*lacZ*ΔM15 Δ(*lacZYA-argF*)*U169 hsdR17*(r_K_^−^ m_K_^+^) λ^−^	35
BW23474	Cloning host for pPB101 and derivatives; Δ*lac-169 robA1 cre C510 hsdR514 endA recA1* Δ*uidA*::*pir-116*	36
Plasmids		
pHA101	pLT06 plasmid with *oriT* from pHOU2 inserted at PstI	21
pCAT28	Shuttle vector; pUC and pAMβ1 origins; confers chloramphenicol resistance	M. Rodrigues and K. Palmer
pFL102	pCAT28 containing 882-bp EcoRI/BamHI-digested *chlR* ORF and promoter region	This study
pFL103	pCAT28 containing 3,673-bp EcoRI/BamHI-digested *efrEF* ORF and promoter region	This study
pPB101	pTCV-lac-cat; expression vector for Gram-positive bacteria; confers kanamycin, erythromycin, and chloramphenicol resistance	21
pFL201	pPB101 with 114-bp EcoRI/BamHI-digested *efrEF* promoter region	This study
pFL202	pPB101 with 114-bp EcoRI/BamHI-digested *chlR* promoter region	This study
pFL203	pPB101 with 98-bp EcoRI/BamHI-digested *efrEF* promoter region	This study
pFL204	pPB101 with 98-bp EcoRI/BamHI-digested *efrEF* promoter region with modified motif CAGCTAC	This study

### Susceptibility testing.

Unless otherwise noted, the CHG used in this study was commercially available Hibiclens (referred to here as H-CHG). The H-CHG MIC was determined in BHI broth using broth microdilution. The MIC was defined as the lowest concentration of H-CHG that inhibited visible cell growth. MIC values were independently confirmed using chlorhexidine digluconate (Sigma). For all experiments in this study, 1× MIC refers to the E. faecalis V583 H-CHG MIC determined by broth microdilution.

### Growth kinetic assays with H-CHG.

E. faecalis V583 growth was monitored by recording the optical density of the cultures at 600 nm (OD_600_) using a spectrophotometer. An overnight culture was diluted to an OD_600_ of 0.01 in BHI broth and incubated at 37°C with agitation at 100 rpm. At mid-log phase (OD_600_, between 0.4 and 0.5), 25 ml of culture was split into flasks with prewarmed medium with or without H-CHG such that concentrations of 1× MIC and 1/2× MIC were attained or no H-CHG was present (control). Growth was then monitored at 15-min intervals for the first half hour and 30-min intervals for the subsequent 3 h.

### Transcriptomic analysis.

Total RNA was extracted from the E. faecalis V583 cultures after 15 min exposure to 1× MIC H-CHG or no H-CHG. Briefly, 10 ml culture was transferred to 20 ml RNA Protect Bacteria reagent (Qiagen) and incubated at room temperature for 10 min. Cells were then pelleted by centrifugation at 11,000 × *g*, resuspended in IHB-1 buffer (50 mM glucose, 25 mM Tris, 10 mM EDTA, pH 8.0) supplemented with 125 μl of a 50-mg/ml lysozyme stock and 25 μl of a 2.5-kU/ml mutanolysin stock, and incubated at 37°C for 20 min. Total RNA was isolated by RNA Bee (Tel-Test) extraction following the manufacturer's protocol. RNA was dissolved in 50 μl RNase-free water (Ambion). RNA samples were treated with RNase-free DNase I (Roche) to remove contaminating DNA and purified using a Qiagen RNeasy kit. DNA contamination was monitored by PCR with primers targeting a 16S rRNA gene (Table S1). RNA integrity was confirmed by visualization of intact 23S and 16S rRNA bands on a 1% agarose gel. RNA was quantified with a NanoDrop spectrophotometer. cDNA was synthesized using SuperScript II reverse transcriptase (Invitrogen) and random hexamers (Qiagen). Three micrograms of cDNA was fragmented with Roche DNase I and 3′ end labeled using a Bioarray terminal labeling kit (Enzo). Labeled, fragmented cDNA was hybridized to custom Affymetrix GeneChips probing the E. faecalis V583 gene sequences (Gilmorea520187F) (15). Processing of Affymetrix GeneChips was performed at the University of Iowa DNA facility. Two independent transcriptome experiments were performed.

### Microarray data analysis.

Microarray data were processed by the bioconductor package based on the R statistical programming environment. The .cel files were preprocessed by use of the robust multiarray average (RMA) algorithm, and the processed data were subjected to gene expression analysis by the linear models for microarray data (Limma) package (28). All codes utilized for gene expression analysis followed the Limma user's guide. Statistical analysis was performed utilizing two independent microarray data sets. Genes with a fold change of ≥4 and a false discovery rate-adjusted *P* value of <0.05 were considered further.

### Semiquantitative and quantitative RT-PCR.

Reverse transcriptase PCR (RT-PCR) was performed to confirm select microarray results and to determine whether *efrE* and *efrF* are cotranscribed. For semiquantitative RT-PCR, RNA (100 ng) was used as the template for cDNA synthesis with 250 ng random hexamers and SuperScript II reverse transcriptase (Invitrogen). Five nanograms of the resulting purified cDNA was used as the template for PCR. The housekeeping gene *clpX* was used as a control. Quantitative reverse transcriptase PCR (qRT-PCR) was performed with an AzuraQuant green fast quantitative PCR (qPCR) mix per the manufacturer's recommendations. qRT-PCR experiments were performed independently three times. The *gyrB* housekeeping gene was used as a control, and expression was normalized to the level of expression of this gene. Statistical significance was assessed by the Student *t* test.

### Construction of deletion mutants.

Vector pHA101 (21), a derivative of pLT06 (29), was used to create deletion mutants of E. faecalis V583. Mutants were generated by markerless in-frame deletion as previously described (21). Briefly, ∼1.0-kb regions upstream and downstream of the gene(s) targeted for deletion were amplified by PCR. Products were digested by the restriction enzymes indicated in Table 1 and ligated with pHA101. Plasmid constructs were propagated in E. coli EC1000 with chloramphenicol selection. Plasmids were transformed into E. faecalis V583 cells by electroporation (30). Deletions were generated using temperature shift and *p*-chlorophenylalanine counterselection as previously described (21, 29).

### Complementation of deletion mutants.

Deletion mutant strains were complemented in *trans* using the shuttle vector pCAT28, a derivative of pAT28 (31) that confers chloramphenicol resistance. For complementation, the *chlR* or *efrEF* complete ORFs with predicted promoter regions were amplified by PCR, treated with the restriction enzymes indicated in Table 1 and S1, and ligated into pCAT28. Plasmids were propagated in E. coli DH5α and electroporated into E. faecalis.

### Primer extension.

Total RNA was obtained as described above. Primer extension was performed using 6-carboxyfluorescein-labeled primers as previously described (32). DNA fragment analysis was processed at the University of Oklahoma Health Sciences Center Laboratory for Genomics and Bioinformatics. Data were analyzed by Peak Scanner software (version 1.0; Thermo Fisher). The size of the most abundant cDNA product was used to determine the transcription start site.

### Viability assay.

Broth cultures were adjusted to an OD_600_ of 0.3 and serially diluted in phosphate-buffered saline (PBS). Ten microliters of each dilution was spotted on agar plates containing different concentrations of H-CHG. Colonies were counted after overnight incubation at 37°C. Counts of colonies of between 20 and 200 were taken into consideration and normalized.

### β-Galactosidase assay.

The putative promoter regions of the *efrEF* operon and the *chlR* gene were amplified using PCR. Products were digested by EcoRI and BamHI and ligated into pPB101 (21). pPB101 and derivatives were propagated in E. coli BW23474 and then transformed into E. faecalis strains by electroporation. For the β-galactosidase assay, a qualitative assay was used. Stationary-phase cultures were adjusted to an OD_600_ of 0.3 and diluted in PBS buffer. Ten microliters of each dilution was spotted on BHI agar plates supplemented with 5-bromo-4-chloro-3-indolyl-β-d-galactopyranoside (X-Gal) and different concentrations of H-CHG.

### Accession number(s).

The microarray data have been deposited in the EMBL-EBI data bank (www.ebi.ac.uk/arrayexpress) under accession number E-MTAB-5181.

## Supplementary Material

Supplemental material
