# Supplementary material for: EfrEF and the Transcription Regulator ChlR Are Required for Chlorhexidine Stress Response in Enterococcus faecalis V583
Source: Antimicrob Agents Chemother. 2018 May 25;62(6):e00267-18. doi: 10.1128/AAC.00267-18 (PMC5971576; doi:10.1128/AAC.00267-18)
Supplement: Supplemental material [file supp_62_6_e00267-18__index.html]

Supplemental material 

# EfrEF and the Transcription Regulator ChlR Are Required for Chlorhexidine Stress Response in Enterococcus faecalis V583

## Supplemental material

- Supplemental file 1 -

  Supplemental Figures S1 and S2 and Table S1

  PDF, 1.1M
- Supplemental file 2 -

  Data Set S1

  XLSX, 19K
