# Supplementary material for: EfrEF and the Transcription Regulator ChlR Are Required for Chlorhexidine Stress Response in Enterococcus faecalis V583
Source: Antimicrob Agents Chemother. 2018 May 25;62(6):e00267-18. doi: 10.1128/AAC.00267-18 (PMC5971576; doi:10.1128/AAC.00267-18)
Supplement: Supplemental material [file AAC.00267-18_zac006187201s1.pdf]

Fig. S1

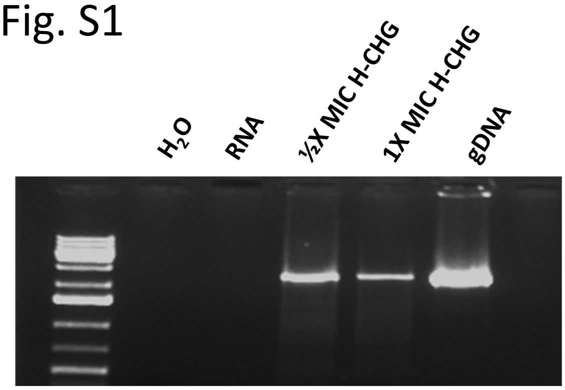

Figure S1. *efrE* and *efrF* are co-transcribed. Primers nested within *efrE* and *efrF* were used in PCR reactions to test whether *efrE* and *efrF* are co-transcribed. RNA was harvested from *E. faecalis* V583 after 15 mins exposure to 1/2X MIC H-CHG or 1X MIC H-CHG. cDNA was synthesized using 100 ng RNA, and 5 ng was used for subsequent PCR. PCR templates used were water or 100 ng RNA (negative controls), purified genomic DNA (gDNA; positive control), or 5 ng cDNA.

Fig. S2

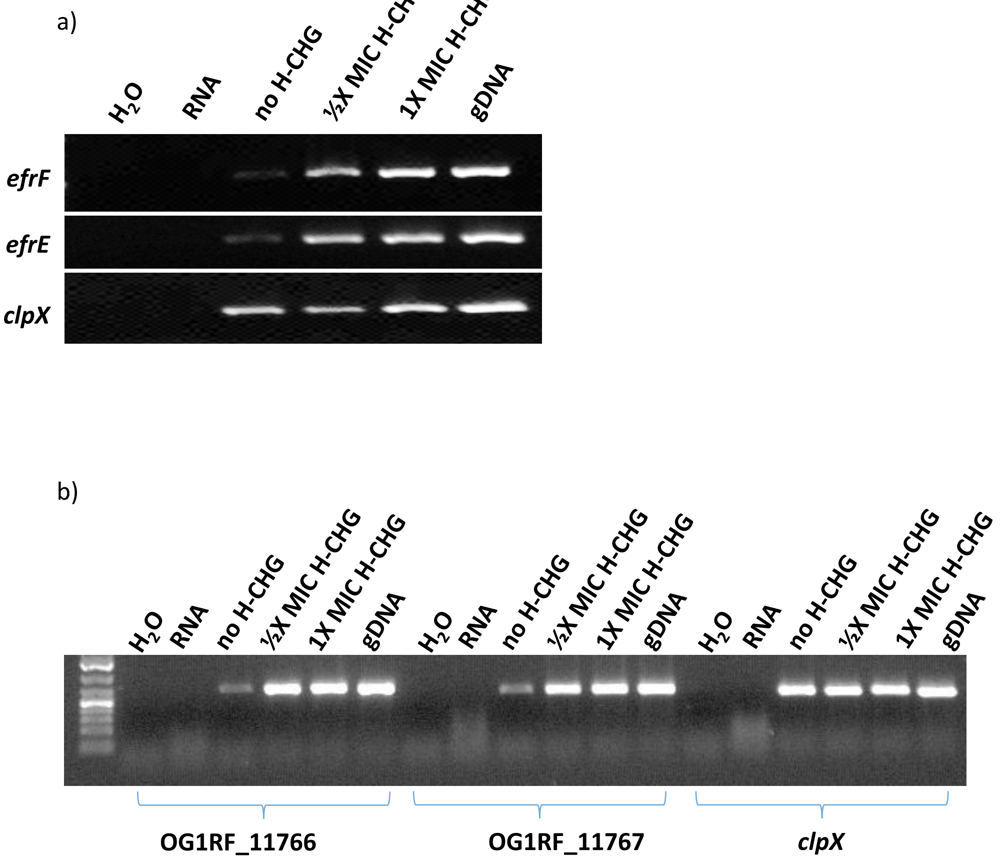

Figure S2. Semi-quantitative RT-PCR confirm H-CHG-dependent up-regulation of *efrEF*. Primers were designed to amplify ~500 bp and 600 bp internal regions of *efrE* and *efrF,* respectively. RNA was harvested from *E. faecalis* V583 (panel a) or *E. faecalis* OG1RF (panel B) after 15 mins exposure to no CHG, 1/2X MIC H-CHG or 1X MIC H-CHG. With 100 ng of RNA as template, cDNA was synthesized using Supercript II reverse transcriptase (Invitrogen) and random hexamers, as outlined by the manufacturer. Then 5 ng cDNA was used for subsequent PCR. PCR templates used were water or 100 ng RNA (negative controls), purified genomic DNA (gDNA; positive control), or 5 ng cDNA. *clpX* was amplified as a control gene. The identical primers set were used for (a) V583 and (b) OG1RF.

EF2226 and EF2227 Knockout	TAACCGGAATTCATGCTCTCCGACAACGATGC<br>TCGTAACTGCAGCAAGGGTTCCTGCGATTTGC<br>TCGTAACTGCAGACTGTCACAGAAAATCGAACAAGC<br>TAACGCGGATCCAATTAAAAGAGCACTATCGCCATGG
EF2226 and EF2227 complementation	TAACCGGAATTCACTGTTCCCTCCATCAAAAAGTTTAGC<br>TAACGCGGATCCTTAAGTGGTTTGAAATTGAC
EF2227 Knockout	TAACCGGAATTCCCGTTGCTTCCTTGAATCGC<br>TCGTAACTGCAGGACCATAAAAGATTGGATGCTCAGG<br>TCGTAACTGCAGACTGTCACAGAAAATCGAACAAGC<br>TAACGCGGATCCAATTAAAAGAGCACTATCGCCATGG
MerR Knock out	TAACCGGAATTCTGACCAATAACTTGTTCGACGC<br>TCGTAACTGCAGGGTTGGATTGAAATTTTAGGGGAAA<br>TCGTAACTGCAGCGTCCCAATAACGAATCGTGG<br>TAACGCGGATCCGTGCCAATTTGTAGGGTGCC
MerR complementation	TAACCGGAATTCTTACTTTTCCCCTAAA<br>TAACGCGGATCCGAAATTTCCTCCTTAAACAA
Co-transcription of EF2226 and EF2227	CACAAGGCGGGTCCAACTAT<br>TACTGCCGCCAATTAGGAGC
RT-PCR testing EF2226 and EF2227 expression	TCGCTTGGGTAACATTGGCT<br>CACCAACAATCGCCGTTGTT<br>ACAATCACCAAGTGGGCGAT<br>GCGGTAGAAATCGGCTCAGA
Primer extension EF2226	AAACAGATGGTTAAAGTTGTCGC
EF2226 promoter reporter	TAACCGGAATTCACTGTTCCCTCCATCAAAAAGTTTAGC<br>TAACGCGGATCCGAAATTTCCTCCTTAAACAA
MerR promoter reporter	TAACGCGGATCCACTGTTCCCTCCATCAAAAAGTTTAGC<br>TAACCGGAATTCGAAATTTCCTCCTTAAACAA
MerR binding site control	TAACCGGAATTCACTGTTCCCTCCATCAAAAAGTTTAGC<br>TACTAGGGATCCAATACCGTTTTAGCTGTTACA
EfrEF complemetation	cttttattgcGGTACCGAGCTCGAATTC<br>ttgatggagggaacagtCGGGGATCCTCTAGAGTC<br>aggatccccgACTGTTCCCTCCATCAAAAAG<br>gctcggtaccGCAATAAAAGAACTGGAACG
clpX primers	CGCACACTTTCTGTTGCTG<br>CCATCAAATGCTCCACCAAC
16S E. faecalis primers	CATGCAAGTCGAACGCTTCT<br>CCATATATCTACGCATTTCAC
Gyrase B primers	ATCATCGACACTCACTCGCG<br>TCAACGTTACAAAGGGTTGGG

Table S1. Primers used in this study
